# The Impact of Diabetes Mellitus and Corresponding HbA1c Levels on the Future Risks of Cardiovascular Disease and Mortality: A Representative Cohort Study in Taiwan

**DOI:** 10.1371/journal.pone.0123116

**Published:** 2015-04-13

**Authors:** Yun-Yu Chen, Yenn-Jiang Lin, Eric Chong, Pei-Chun Chen, Taz-Fan Chao, Shih-Ann Chen, Kuo-Liong Chien

**Affiliations:** 1 Division of Cardiology, Department of Medicine, Taipei Veterans General Hospital, Taipei, Taiwan; 2 Institute of Epidemiology and Preventive Medicine College of Public Health, National Taiwan University, Taipei, Taiwan; 3 School of Medicine, Institute of Clinical Medicine, and Cardiovascular Research Center, National Yang-Ming University, Taipei, Taiwan; 4 Department of Cardiology, Jurong Health Private Limited, Singapore, Singapore; National University of Singapore, SINGAPORE

## Abstract

**Background:**

This study explored the relationship between the glycated hemoglobin (HbA1c) level in patients with or without diabetes mellitus and future risks of cardiovascular disease and death.

**Methods:**

Based on a national representative cohort, a total of 5277 participants (7% with diabetes) were selected from Taiwan's Triple High Survey in 2002. The comorbidities, medication usages, and outcomes of cardiovascular disease and death, were extracted from the Taiwan’s National Health Insurance Research Database and National Death Registry.

**Results:**

After a median follow-up of 9.7 years, participants with diabetes had higher incidence of new onset cardiovascular disease (17.9 versus 3.16 cases per 1000 person-years) and death (20.1 versus 4.96 cases per 1000 person-years) than those without diabetes (all P < 0.001). Diabetes showed increased risk of all-cause death after adjusting for all confounders (adjusted hazard ratio [HR]: 2.29, 95% confidence interval [CI]: 1.52-3.45). Every 1% increment of HbA1c was positively associated with the risk of total cardiovascular disease (HR: 1.2, 95% CI: 1.08-1.34) and the risk of death (HR: 1.14, 95% CI: 1.03-1.26) for all participants. As compared to the reference group with HbA1c below 5.5%, participants with HbA1c levels ≥7.5% had significantly elevated future risks of total cardiovascular disease (HR: 1.82, 95% CI: 1.01-3.26) and all-cause death (HR: 2.45, 95% CI: 1.45-4.14).

**Conclusions/Interpretation:**

Elevated HbA1C levels were associated with increased risks of cardiovascular disease and death, the suboptimal glycemic control with HbA1c level over 7.5% (58.5 mmol/mol) was strongly associated with increased risks of cardiovascular disease and all-cause death.

## Introduction

Optimal management of diabetes mellitus is a major concern for healthcare professionals due to its strong association with cardiovascular events (including coronary heart diseases and strokes) and deaths. Glycated hemoglobin (HbA1c) is an established marker for evaluating glucose level at an intermediate term, as it reflects the average plasma glucose control over a period of 2–3 months [1].

Currently, the association between chronic hyperglycemia and macrovascular complications is not well defined. Several observational studies demonstrated that a higher HbA1c level was associated with increased risks of cardiovascular diseases and deaths [2–4]. On the other hand, a meta-analysis study showed that 1% HbA1c reduction was associated with a lowered major cardiovascular risks by glycemic control, but was not associated with lowered stroke and death risks [5]. Growing evidence supports the finding that HbA1c level is an independent risk factor for cardiovascular events, regardless of the diagnosis of diabetes [3,6–9].

In the current guidelines [10], HbA1c of 6.5% is a recommended cut-off point for the diagnosis of diabetes. However, the expert group agreed that there is still a lack of sufficient evidence to make any formal recommendation on the interpretation of HbA1c levels below 6.5%. Therefore, the suitability of HbA1c levels for glycemic control needs to be assessed. The current study aimed to explore the risks of cardiovascular disease and death that are associated with diabetes and HbA1c levels.

## Methods

### Study design and participants

**Fig 1:**
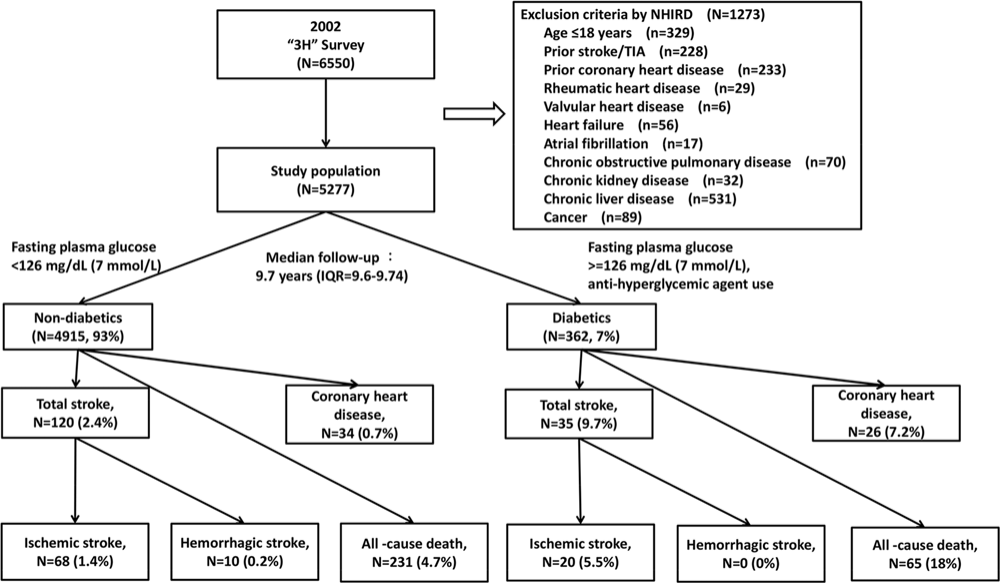
Study flow chart.

This is a population-based retrospective cohort study; participants were selected from the 2002 Taiwan's Triple High Survey. The protocol was reviewed and approved by the Research Ethics Committee of National Taiwan University Hospital. The committee was organized under and operated in accordance with the Good Clinical Practice Guidelines (NTUH-REC Number: 201305044W [Institutional Review Board reference, IRB]). Additionally, we obtained permission of the rights from the National Research Institute for the Department of Health, and the Health Promotion Administration, Ministry of Health and Welfare. This study complies with the categories of exempt, in accordance with the governmental laws and regulations. We excluded individuals who were younger than 18 years or had a stroke, transient ischemic attack, coronary heart disease, chronic kidney disease, chronic liver disease, rheumatic heart disease, valvular heart disease, heart failure, atrial fibrillation, chronic obstructive pulmonary disease, or cancer prior to the enrollment date of Triple High Survey (Fig 1).

### Databases

#### National Health Interview Survey, 2001

The Taiwan National Health Interview Survey (NHIS, 2001) was a general survey conducted from August 2001 to January 2002 that was approved by both ethics committees/IRBs of the National Research Institute for the Department of Health and the Health Promotion Administration, Ministry of Health and Welfare. Citizens aged over 15 years who were willing to sign the informed consent form were included. The target population of the original survey was 22287976 individuals whose households were registered in one of the 23 counties or cities in Taiwan. The study sample was collected nationwide via a multi-stage stratified systematic sampling method: a total of 359 towns and districts were classified into 7 regions and the number of neighborhoods in the regions was selected according to the population in the regions. Four households were selected from each neighborhood according to degree of citizenship, age, and sex. A total of 26680 participants were personally interviewed by well-trained interviewers and all participants undertook written, informed consent for survey. The informed consent forms were well-documented and recorded in the computer system by the National Research Institute for the Department of Health and the Health Promotion Administration, Ministry of Health and Welfare.

#### Taiwan’s Hypertensive, Hyperglycemia, Hyperlipidemia Survey, 2002

Taiwan’s Hypertensive, Hyperglycemia, Hyperlipidemia Survey (Triple High Survey, 2002) was based on NHIS (2001) from March 2002 to October 2002; this was the second nationwide health survey designed for national population samples [11]. The Triple High Survey was also approved by the ethics committee/IRB of the Health Promotion Administration, Ministry of Health and Welfare. Participants who were in the initial survey of NHIS (2001) were randomly assigned to the Triple High Survey (2002) if the signed informed consent form was available. In other words, the inclusion criteria of the Triple High Survey (2002) were similar to the Taiwan National Health Interview Survey (2001). We could only access participants who signed the informed consent and the data linkage agreement form in the original cohort of the Triple High Survey. A total of 6550 participants underwent biochemical measurements that were available for analyses and HbA1c was measured in all participants.

#### Taiwan’s National Health Insurance Research Database

The Taiwan’s National Health Insurance (NHI) program enrolled 23 million people, which covered 99% of the country’s population. The National Health Insurance Research Database (NHIRD) contained data on utilization of all NHI resources, including out-patient visits, hospital care, prescribed medications, and National Death Registry. The insurance claim database can be used for studies of natural diseases and clinical research in real-world clinical settings.

#### Ascertainment of underlying diseases and medications

The Collaboration Center of Health Information Application, Ministry of Health and Welfare, Taiwan, provided the entire datasets used in our study. Diabetes, hypertension, dyslipidemia, and hyperuricemia were defined according to World Health Organization (WHO) definitions based on clinical history, medications, or biomarkers in the Triple High Survey. In order to improve the accuracy of disease diagnoses, the medications used in NHIRD were also analyzed. History of stroke among first-degree relatives was reflected in the Triple High Survey.

NHIRD was used to identify underlying illnesses and associated medication usages aside from diabetes, hypertension, dyslipidemia, and hyperuricemia. The International Classification of Diseases, Ninth Revision—Clinical Modification (ICD9-CM) codes were ascertained between 2000–2002; the diagnoses must have recorded twice in out-patient records or at least once in in-patient records. Total stroke was defined as combination of ischemic stroke (433–434), hemorrhagic stroke (430–432) and other unknown strokes (435–438). The identification of ICD9-CM for ischemic stroke was according to the definition by Cheng [12]. Total cardiovascular disease was defined as coronary heart disease (acute myocardial infarction and unstable angina, 410–411) or stroke. The selection and grouping of medications were according to the guidelines of the Anatomical Therapeutic Chemical (ATC) classification system by the WHO.

Diabetes was confirmed if participants had a confirmed history of the disease, were being treated with anti-hyperglycemic agents, or had fasting glucose ≥7 mmol/L (126 mg/dL) (Fig 1). Hypertension was confirmed if participants had a confirmed history of the disease, were being treated with anti-hypertensive agents, or had systolic blood pressure (SBP) >140 mm Hg or diastolic blood pressure (DBP) >90 mm Hg. Dyslipidemia was confirmed if participants had a history of the condition, were being treated with lipid lowering drugs, or had total cholesterol >5.18 mmol/L (200 mg/dL), low-density lipoprotein cholesterol (LDL-C) >3.37 mmol/L (130 mg/dL), high-density lipoprotein cholesterol (HDL-C) <1.03 mmol/L (40 mg/dL), or triglyceride ≥1.69 mmol/L (150 mg/dL). Hyperuricemia was confirmed if participant had a history of the condition, were being treated with uric acid lowering agents, or had uric acid >416 μmol/L (7 mg/dL) for men or >357 μmol/L (6 mg/dL) for women.

### The methodology of examination for measures

The blood sampling was performed after a 12-hour fasting period. HbA1c was measured using high performance liquid chromatography, quality control was assured under the criteria by the National Glycohemoglobin Standardization Program. The variability was within ±0.5% HbA1c and between-laboratory coefficients of variations were <5%. LDL-C was measured by the formula of "total cholesterol—HDL-C—(Triglyceride/5)". Total cholesterol and triglyceride were examined by colorimetry (Bucolo method), and HDL-C was examined by electrophoresis. Creatinine and uric acid were examined by immunoturbidimetric method.

An adjusted mercury sphygmomanometer was used to measure blood pressure according the clinical practice guideline for hypertension by the American Society of Hypertension. Participants were asked to rest for at least 30 minutes before measurement of blood pressure. Waist circumference was measured at the mid-distance between the last rib margin and iliac crest during exhalation. A recall value of waist circumference was recorded for physically disabled or pregnant participants.

### End points

The primary endpoint was the incidence of total cardiovascular disease, including: stroke (ischemic/hemorrhagic) and coronary heart disease, whichever came first. The secondary end-point was all-cause death. Total cardiovascular disease was tracked using the NHIRD according to the ICD9-CM codes during hospitalizations from 2003 to 2011. Death was confirmed by referencing the Taiwan's National Death Registry. The follow-up period ended when the subjects developed stroke, death, or lived beyond 31 December 2011.

### Statistical methods

The normally distributed continuous variables were compared using the Student's t-test, whereas the non-normally distributed variables were compared using the Mann-Whitney U test. Frequencies were compared with the chi-square test. The incidence rates of cardiovascular events were calculated as the number of cases per 1000 person-years of follow-up. Participants were divided into 4 groups of HbA1c in the analyses (<5.5% [36.5 mmol/mol], 5.5–6.4% [36.5–46.4 mmol/mol], 6.5–7.4% [47.5–57.4 mmol/mol], and ≥7.5% [58.5 mmol/mol]).

The Cox proportional-hazards regression was used to compare the hazard ratios (HRs) for the outcomes. Potential confounders were adjusted via three models. Model 1: age group (<45, 45–54, 55–64, 65–74, ≥75 years), and sex; Model 2: factors in Model 1 plus family history of stroke, waist circumference (per 1 cm), systolic blood pressure (per 1 mm Hg), triglyceride (per 1 mg/dL), HDL-C (per 1 mg/dL), uric acid (per 1 mg/dL), and creatinine (per 1 mg/dL); Model 3: factors in Model 2 plus anti-hyperglycemic drugs (only in diabetic group), lipid-lowering drugs, anti-hypertensive drugs, anti-platelet drugs, and anti-acid agents.

The level of statistical significance was set at a 2-tailed alpha level <0.05. Analyses were performed with SAS version 9.3 (SAS Institute, Cary, NC).

## Results

### Baseline characteristics of this study

This study involved 5277 participants who met the inclusion criteria, the dataset included 362 diabetic participants (7%) and 4915 non-diabetic participants (93%) under the median follow-up period of 9.7 years (interquartile range: 9.6–9.74 years; Fig 1). The baseline characteristics of our selected samples was similar to the original population who aged more than 18 years (6532 participants; Table 1). Among the 5277 study participants, mean age was 42±15 years in non-diabetic group, and 56±14 years in diabetic group (P<0.001). The male proportion was similar between non-diabetic group (46.5%) and diabetic group (50.8%; P> 0.05). The diabetic group had poorer glycemic control in terms of HbA1c and lipid profile (P < 0.001).

**Table 1:** Baseline characteristics of participants in the diabetic and non-diabetic groups.

Variables	Original population (N = 6532)	Study population (N = 5277)	Non-diabetis (N = 4915)	Diabetis (N = 362)	P-value
Age, years	43±17	43±16	42±15	56±14	<0.001
Male gender (%)	3137 (48%)	2471 (46.8%)	2287 (46.5%)	184 (50.8%)	0.11
1^st^ degree relatives with stroke (%)	1373 (21%)	1039 (19.7%)	924 (18.8%)	115 (31.8%)	<0.001
**Underlying diseases**					
Hypertension (%)	2374 (36.3%)	1744 (33%)	1497 (30.5%)	247 (68.2%)	<0.001
Dyslipidemia (%)	3136 (48%)	2531 (48%)	2262 (46%)	269 (74.3%)	<0.001
Hyperuricemia (%)	1846 (28.3%)	1430 (26.9%)	1313 (26.7%)	107 (29.6%)	0.24
**Medication uses**					
Statins (%)	250 (3.8%)	148 (2.8%)	100 (2%)	48 (13.3%)	<0.001
Fibrates (%)	121 (1.9%)	76 (1.4%)	42 (0.9%)	34 (9.4%)	<0.001
Insulin (%)	49 (0.8%)	20 (0.4%)	0 (0%)	20 (5.5%)	<0.001
Oral hyperglycemic agents (%)	350 (5.4%)	216 (4.1%)	0 (0%)	216 (59.7%)	<0.001
Anti-hypertensive agents (%)	1947 (29.8%)	1364 (25.8%)	1149 (23.4%)	215 (59.4%)	<0.001
Anti-platelet agents (%)	805 (12.3%)	473 (9%)	400 (8.1%)	73 (20.2%)	<0.001
Anti-acid agents (%)	198 (3%)	146 (2.8%)	117 (2.4%)	29 (8%)	<0.001
**Biochemical measures**					
HbA1c, % (mmol/mol)	5.36 (35)±1.06	5.33 (34.6)±1.01	5.15 (32.7)±0.54	7.72 (60.9)±2.16	<0.001
Fasting glucose, mmol/L	5.24±1.59	5.2±1.52	4.91±0.52	9.11±3.67	<0.001
Total cholesterol, mmol/L	4.73±0.98	4.76±0.97	4.71±0.93	5.35±1.34	<0.001
Triglyceride, mmol/L	1.43±0.95	1.42±0.95	1.37±0.87	2.17±1.5	<0.001
HDL-C, mmol/L	1.43±0.39	1.44±0.39	1.44±0.38	1.34±0.5	<0.001
LDL-C, mmol/L	2.97±0.71	3±7	2.97±0.68	3.34±0.82	<0.001
Systolic blood pressure, mm Hg	116±18	115±17.8	114±17.4	128±19.3	<0.001
Diastolic blood pressure, mm Hg	74.9±11.3	74.9±11.3	74.5±11.2	80.1±11.1	<0.001
Uric acid, μmol/L	378±107	375±106	374±105	387±121	<0.01
Creatinine, μmol/L	80.4±28.3	79.6±19.5	78.7±17.7	84±35.4	<0.01
Waist circumference, cm	79.5±11.1	79.8±11.4	79±10.9	87.2±10.8	<0.001

Data were presented as number (%), or mean±standard deviation.

### Diabetes as a risk of cardiovascular disease and all-cause mortality

Table 2 compares the cumulative and mean incidences between non-diabetics and diabetics, and separates the incidences by HbA1c levels for all of the 5277 participants. Hemorrhagic strokes were rare in both the diabetic and non-diabetic groups, therefore, we did not consider hemorrhagic stroke in this study (Fig 1). Diabetics and higher HbA1c level had increased crude incidence rates of stroke, coronary heart disease, and all-cause death: the mean incidence of ischemic stroke was 6.4 per 1000 person-years in diabetics, as compared with 1.47 per 1000 person-year in non-diabetics. The incidence of coronary heart disease was 8.21 per 1000 person-years in diabetics, as compared with 0.73 per 1000 person-years in non-diabetics. The incidence of all-cause death was 20.1 per 1000 person-years in diabetics, as compared with 4.96 per 1000 person-years in non-diabetics. However, diabetes was only statistically significant to all-cause death instead of cardiovascular diseases after controlling for all confounders (HR: 2.29, 95% CI: 1.52–3.45; Table 3).

**Table 2:** Various incidence rates and effect sizes of cardiovascular disease and death by using univariable analysis .

Variables	Total numbers	Person-years	Ischemic stroke				Total stroke			
			Event (%)	Incidence rate (per 1000 PY)	Crude HR (95% CI)	P-value	Event (%)	Incidence rate (per 1000 PY)	Crude HR (95% CI)	P-value
**Non-diabetis**	4915	46266	68 (1.38%)	1.47	1 (Reference)	-	120 (2.44%)	2.59	1 (Reference)	-
**Diabetis**	362	3125	20 (5.52%)	6.4	4.23 (2.69–7.29)	<0.001	35 (9.67%)	11.2	4.38 (3.00–6.38)	<0.001
**HbA1c (1% increment)**	5277	49391	88 (1.67%)	-	1.47 (1.34–1.61)	<0.001	155 (2.94%)	-	1.43 (1.33–1.54)	<0.001
**HbA1c group**										
**<5.5% (36.5 mmol/mol)**	3899	36904	34 (0.87%)	0.92	1 (Reference)		70 (1.80%)	1.90	1 (Reference)	-
**5.5–6.4 (36.5–46.4)**	1022	9367	33 (3.23%)	3.52	3.85 (2.39–6.22)	<0.001	52 (5.09%)	5.55	2.95 (2.06–4.22)	<0.001
**6.5–7.4 (47.5–57.4)**	173	1541	9 (5.20%)	5.84	6.46 (3.10–13.5)	<0.001	13 (7.51%)	8.44	4.52 (2.50–8.16)	<0.001
**≥7.5 (58.5)**	183	1579	12 (6.56%)	7.60	8.41 (4.36–16.3)	<0.001	20 (10.9%)	12.7	6.79 (4.13–11.2)	<0.001
			**Coronary heart disease**				**Total cardiovascular disease**			
**Non-diabetis**	4915	46507	34 (0.69%)	0.73	1 (Reference)	-	146 (2.97%)	3.16	1 (Reference)	-
**Diabetis**	362	3169	26 (7.18%)	8.21	11.4 (6.86–19.1)	<0.001	55 (15.2%)	17.9	5.78 (4.24–7.88)	<0.001
**HbA1c (1% increment)**	5277	49675	60 (1.14%)	-	1.57 (1.43–1.72)	<0.001	201 (3.81%)	-	1.47 (1.38–1.56)	<0.001
**HbA1c group**										
**<5.5% (36.5 mmol/mol)**	3899	37026	18 (0.46%)	0.49	1 (Reference)		86 (2.21%)	2.33	1 (Reference)	-
**5.5–6.4 (36.5–46.4)**	1022	9488	20 (1.96%)	2.11	4.36 (2.31–8.24)	<0.001	67 (6.56%)	7.18	3.10 (2.25–4.27)	<0.001
**6.5–7.4 (47.5–57.4)**	173	1556	5 (2.89%)	3.21	6.73 (2.50–18.1)	<0.001	17 (9.83%)	11.2	4.86 (2.89–8.18)	<0.001
**≥7.5 (58.5)**	183	1605	17 (9.29%)	10.6	22.3 (11.5–43.2)	<0.001	31 (16.9%)	20.1	8.80 (5.84–13.3)	<0.001
			**All-cause death**							
**Non-diabetis**	4915	46619	231 (4.70%)	4.96	1 (Reference)	-				
**Diabetis**	362	3238	65 (18.0%)	20.1	4.1 (3.11–5.39)	<0.001				
**HbA1c (1% increment)**	5277	49857	296 (5.61%)	-	1.34 (1.26–1.42)	<0.001				
**HbA1c group**										
**<5.5% (36.5 mmol/mol)**	3899	37087	147 (3.77%)	3.96	1 (Reference)	-				
**5.5–6.4 (36.5–46.4)**	1022	9544	90 (8.81%)	9.43	2.39 (1.84–3.10)	<0.001				
**6.5–7.4 (47.5–57.4)**	173	1575	24 (13.9%)	15.2	3.88 (2.52–5.97)	<0.001				
**≥7.5 (58.5)**	183	1651	35 (19.1%)	21.2	5.39 (3.73–7.80)	<0.001				

**Table 3:** Various models for the effect sizes according to diabetes and 1% increment of HbA1c.

Outcomes	Models	Non-diabetes (reference) versus diabetes		1% increment of HbA1c					
		All participants (N = 5277)		All participants (N = 5277)		Non-diabetic group (N = 4915)		Diabetic group (N = 362)	
		HR (95% CI)	P-value	HR (95% CI)	P-value	HR (95% CI)	P-value	HR (95% CI)	P-value
**Ischemic stroke**	**Model 1**	1.76 (1.06–2.92)	0.03	1.28 (1.15–1.44)	<0.001	1.50 (1.13–1.99)	<0.01	1.23 (1.00–1.50)	0.04
	**Model 2**	1.47 (0.87–2.50)	0.15	1.27 (1.12–1.44)	<0.001	1.45 (1.08–1.93)	0.01	1.25 (1.01–1.54)	0.04
	**Model 3**	1.34 (0.57–3.12)	0.51	1.29 (1.12–1.49)	<0.001	1.40 (1.04–1.87)	0.02	1.26 (1.00–1.59)	0.04
**Total stroke**	**Model 1**	1.80 (1.23–2.63)	<0.001	1.24 (1.14–1.36)	<0.001	1.22 (0.93–1.60)	0.15	1.20 (1.03–1.40)	0.02
	**Model 2**	1.59 (1.06–2.38)	0.02	1.23 (1.11–1.36)	<0.001	1.17 (0.87–1.56)	0.30	1.22 (1.04–1.44)	0.02
	**Model 3**	1.11 (0.54–2.30)	0.78	1.20 (1.06–1.36)	<0.001	1.34 (0.85–1.51)	0.39	1.22 (1.02–1.45)	0.03
**Coronary heart disease**	**Model 1**	4.82 (2.86–8.12)	<0.001	1.41 (1.27–1.58)	<0.001	1.11 (0.63–1.94)	0.71	1.19 (1.00–1.42)	0.04
	**Model 2**	3.08 (1.75–5.42)	<0.001	1.33 (1.17–1.52)	<0.001	1.47 (0.49–1.71)	0.79	1.25 (1.02–1.52)	0.03
	**Model 3**	1.79 (0.62–5.15)	0.28	1.23 (1.04–1.47)	<0.01	0.88 (0.46–1.67)	0.69	1.31 (1.06–1.61)	0.01
**Total cardiovascular disease**	**Model 1**	2.41 (1.76–3.30)	<0.001	1.29 (1.20–1.40)	<0.001	1.15 (0.89–1.49)	0.30	1.19 (1.05–1.34)	<0.01
	**Model 2**	1.93 (1.38–2.70)	<0.001	1.26 (1.15–1.37)	<0.001	1.06 (0.80–1.40)	0.69	1.22 (1.07–1.39)	<0.01
	**Model 3**	1.33 (0.73–2.43)	0.34	1.20 (1.08–1.34)	<0.001	1.05 (0.79–1.38)	0.75	1.24 (1.08–1.42)	<0.01
**All-cause death**	**Model 1**	1.79 (1.36–2.38)	<0.001	1.15 (1.06–1.24)	<0.001	1.09 (0.87–1.35)	0.46	1.03 (0.91–1.16)	0.68
	**Model 2**	1.63 (1.21–2.20)	<0.001	1.12 (1.03–1.22)	<0.01	1.08 (0.87–1.35)	0.49	1.02 (0.89–1.17)	0.78
	**Model 3**	2.29 (1.52–3.45)	<0.001	1.14 (1.03–1.26)	0.02	1.09 (0.87–1.36)	0.47	1.05 (0.92–1.21)	0.46

Model 1: age group (<45, 45–54, 55–64, 65–74, ≥75 years), and sex; Model 2: factors in Model 1 plus family history of stroke, waist circumference (per 1 cm), systolic blood pressure (per 1 mm Hg), triglyceride (per 1 mg/dL), HDL-C (per 1 mg/dL), uric acid (per 1 mg/dL), and creatinine (per 1 mg/dL); Model 3: factors in Model 2 plus anti-hyperglycemic drugs (only in diabetic group), lipid-lowering drugs, anti-hypertensive drugs, anti-platelet drugs, and anti-acid agents.

### Impact of HbA1c on the risk of stroke and all-cause mortality

By analyzing all 5277 participants, we observed a positive association with the future risks of total cardiovascular diseases and death for every 1% increment of HbA1c (Table 3). The adjusted HRs were 1.29 for ischemic stroke (95% CI: 1.12–1.49), 1.23 for coronary heart disease (95% CI: 1.04–1.47), 1.2 for total cardiovascular disease (95% CI: 1.08–1.34),and 1.14 for all-cause death (95% CI: 1.03–1.26). Every 1% increment of HbA1c was associated with increased risk of ischemic stroke for both non-diabetics and diabetics (adjusted HR: 1.4, 95% CI: 1.04–1.87 for non-diabetics, and adjusted HR: 1.26, 95% CI: 1–1.59 for diabetics). But HbA1c increments were not significantly associated with the risk of all-cause death by diabetes status, and HbA1c increments were not associated with total stroke, coronary heart disease, and cardiovascular disease in non-diabetics.

By taking HbA1c less than 5.5% as a reference value, we observed a dose-response association of elevated HbA1c levels on the HRs for ischemic stroke, total stroke, coronary heart disease, total cardiovascular disease, and all-cause death after adjusting for age, sex, family history of stroke, and essential biochemical data in all participants (Model 2; Table 4). After adjusting for medications (Model 3), the dose-response association of elevated HbA1c levels was also observed for ischemic stroke, total cardiovascular disease, and death risks. HbA1c levels ≥7.5% significantly increased the risks of ischemic stroke, coronary heart disease, total cardiovascular disease, and death compared to the reference HbA1c group (HR: 2.75, 95% CI: 1.12–6.73 for ischemic stroke, HR: 3.09, 95% CI: 1.18–8.13 for coronary heart disease, HR: 1.82, 95% CI: 1.01–3.26 for total cardiovascular disease, and HR: 2.45, 95% CI: 1.45–4.14 for all-cause death).

**Table 4:** Various models for the effect sizes according to HbA1c levels.

Outcomes	Models	HbA1c group				P trend
		<5.5% (<36.5 mmol/mol)	5.5–6.4 (36.5–46.4)	6.5–7.4 (47.5–57.4)	≥7.5 (≥58.5)	
		N = 3899	N = 1022	N = 173	N = 183	
		HR (95% CI)	HR (95% CI)	HR (95% CI)	HR (95% CI)	
**Ischemic stroke**	**Model 1**	1 (Reference)	1.62 (0.99–2.65)	2.28 (1.09–4.81)	3.01 (1.54–5.86)	<0.001
	**Model 2**	1 (Reference)	1.53 (0.93–2.51)	1.91 (0.87–4.21)	2.63 (1.31–5.26)	0.04
	**Model 3**	1 (Reference)	1.52 (0.92–2.51)	1.99 (0.87–4.52)	2.75 (1.12–6.73)	0.04
**Total stroke**	**Model 1**	1 (Reference)	1.29 (0.89–1.86)	1.59 (0.87–2.89)	2.49 (1.51–4.13)	<0.001
	**Model 2**	1 (Reference)	1.32 (0.86–1.81)	1.44 (0.77–2.70)	2.18 (1.27–3.73)	0.04
	**Model 3**	1 (Reference)	1.22 (0.84–1.78)	1.32 (0.68–2.59)	1.69 (0.83–3.45)	0.47
**Coronary heart disease**	**Model 1**	1 (Reference)	1.92 (1.00–3.68)	2.59 (0.95–7.05)	8.09 (4.10–16.0)	<0.001
	**Model 2**	1 (Reference)	1.55 (0.80–2.99)	1.92 (0.70–5.28)	4.93 (2.38–10.2)	<0.001
	**Model 3**	1 (Reference)	1.44 (0.74–2.81)	1.45 (0.51–4.29)	3.09 (1.18–8.13)	0.08
**Total cardiovascular disease**	**Model 1**	1 (Reference)	1.36 (0.98–1.88)	1.75 (1.03–2.96)	3.28 (2.16–4.99)	<0.001
	**Model 2**	1 (Reference)	1.24 (0.89–1.72)	1.47 (0.85–2.55)	2.55 (1.63–3.98)	<0.001
	**Model 3**	1 (Reference)	1.20 (0.86–1.67)	1.31 (0.73–2.35)	1.82 (1.01–3.26)	0.04
**All-cause death**	**Model 1**	1 (Reference)	1.11 (0.85–1.45)	1.42 (0.92–2.21)	2.16 (1.48–3.14)	<0.001
	**Model 2**	1 (Reference)	1.12 (0.85–1.48)	1.33 (0.84–2.08)	1.98 (1.32–2.96)	<0.01
	**Model 3**	1 (Reference)	1.13 (0.86–1.49)	1.45 (0.91–2.32)	2.45 (1.45–4.14)	<0.001

Model 1: age group (<45, 45–54, 55–64, 65–74, ≥75 years), and sex; Model 2: factors in Model 1 plus family history of stroke, waist circumference (per 1 cm), systolic blood pressure (per 1 mm Hg), triglyceride (per 1 mg/dL), HDL-C (per 1 mg/dL), uric acid (per 1 mg/dL), and creatinine (per 1 mg/dL); Model 3: factors in Model 2 plus anti-hyperglycemic drugs (only in diabetic group), lipid-lowering drugs, anti-hypertensive drugs, anti-platelet drugs, and anti-acid agents.

## Discussion

### Main findings

Diabetes led to higher incidence of all-cause death with 1.3-fold increase in adjusted risk. Higher HbA1c levels were positively correlated with future risks of stroke, coronary heart disease, and all-cause death, the risks of ischemic stroke increased by 1% increment of HbA1c regardless of diabetes diagnosis. In addition, HbA1c levels ≥7.5% (58.5 mmol/mol) incrementally and significantly increased future risks of ischemic stroke, coronary heart disease, and all-cause death compared to the reference group of HbA1c levels <5.5% (36.5 mmol/mol).

### Comparisons with previous studies

Several studies have demonstrated that elevated HbA1c levels were associated with increased cardiovascular risk and all-cause death, but there were inconsistent risk stratifications according to different HbA1c levels. In the National Integrated Project for Prospective Observation of Non-communicable Disease and its Trends in the Aged study of 5978 Japanese participants [13], HbA1c levels >6% (42 mmol/mol) were associated with increased all-cause death. There was approximately a 20% increased risk of death for every 1% increment of HbA1c.

In studies involving only diabetes patients, such as the Swedish National Diabetes Register cohort (NDR, 18334 diabetes patients) [2], every 1% increment of HbA1c was associated with a 10%-20% increase in HRs for total stroke, total coronary heart disease, and all-cause deaths, a U-shaped risk curve was not observed regarding the cardiovascular disease and all-cause death. On the other hand, in the United Kingdom Practice Research Database study (UK GPRD) of 27965 patients, the results showed a U-shaped association with all-cause death and the lowest HR corresponded to a HbA1c level of 7.5% (58.5 mmol/mol) [14].

In studies involving non-diabetic patients, such as the Atherosclerosis Risk in Communities Study in USA (ARIC, 11092 participants) [3] and Korean Soonchunhyang Stroke Registry (307 ischemic stroke cases and 253 controls) [4], the researchers showed that elevated HbA1c levels (>5.5%-6% [36.5–42 mmol/mol]) were associated with increased incidence of ischemic stroke, coronary heart disease, and all-cause death. In addition, the ARIC study demonstrated a U-shaped association between HbA1c and all-cause mortality.

The current study demonstrated a 10%-30% increase risk of stroke, coronary heart disease, and death for every 1% increment of HbA1c. Importantly, the relationship between HbA1c increments and risks of ischemic stroke was presented in all participants, regardless of diabetes diagnosis. The results from prior studies were comparable with that of our study.

As mentioned previously, the UK GPRD and ARIC studies showed increased risk of all-cause death with both lower and higher HbA1c levels (a U-shaped association) [3,14], which explored the possible risks in intensive glycemic control; however, we did not observe the U-shaped association for the risks of stroke, coronary heart disease, and mortality at the lower levels of HbA1c after adjusting for all confounders. In this study, the cardiovascular and death risks increased significantly with a dose-response association if HbA1c levels ≥7.5% (58.5 mmol/mol), the present results might support the current target levels of HbA1c <7% (53 mmil/mol) by the guideline of American Diabetes Association [15].

### Possible mechanisms and clinical implications for HbA1c

HbA1c reflects the status of chronic hyperglycemia in the previous 2–3 months. Early endothelial dysfunction and progressive vascular inflammation lead to cardiovascular events [16]. Vascular complications appear to be attributable to the glycation of cellular proteins and lipids over the course of many years, leading to atherosclerosis; thus, many macrovascular diseases are closely linked to the adverse consequences of diabetes [17,18].

Glycemic management in diabetes has become more complex, including the concerns about the potential confounders such as blood pressure, lipid levels, obesity, and uncertainties regarding the pleotropic effect of intensive glycemic control or cardiovascular medications [15]. The United Kingdom Prospective Diabetes Study (UKPDS) 10-year follow-up demonstrated that the relative benefit of having been in the group of intensive management policy, resulting in the emergence of statistically significant benefits on cardiovascular disease and all-cause death [19].

In this study, diabetic participants also had higher blood pressure and lipid levels when compared with non-diabetic participants. These could be important explanations for the increased cardiovascular risks in diabetics. In the UKPDS [20], the researchers concluded that every 10 mm Hg increment in systolic pressure was associated with a significant increase in diabetes-related deaths and cardiovascular complications. Furthermore, the mixed dyslipidemia with elevated triglycerides, low-density lipoprotein cholesterol, or both conditions is associated with cardiovascular risks [21].

Recent observational study showed that the selection of insulin was associated with an increased risk of a composite of nonfatal cardiovascular outcomes and all-cause death in diabetic patients as compared with the selection of sulfonylurea [22]. Also, angiotensin-converting enzyme inhibitors were associated with a reduction of mortality in diabetic patients [23]. In the present study, we applied statistical regression via different models to investigate the association of risk factors for exposures and confounders [24]. After adjusting the medications (Model 3), we still observed significant cardiovascular and death risks with dose-response association by increased HbA1c levels, even through the results of cardiovascular disease were attenuated substantially in model 2 and 3, suggesting the evidence that the level of HbA1C remained to be the risk factor for cardiovascular disease and all-cause death.

### Strengths and limitations

To the best of our knowledge, this is the first study representing real-world epidemiologic data in Taiwan for exploring the cardiovascular according to HbA1c levels; however, there are several limitations. First, this study was based on a nationwide census in Taiwan, and we only had one biochemical measure of HbA1c from a single screening in Taiwan's Triple High Survey to elucidate long-term outcomes. Second, we did not use HbA1c as the diagnosis criteria for diabetes. Diabetes was defined according to the criteria of the American Diabetes Association, 1997, as used in the Triple High Survey (2002). Third, we adjusted for waist circumference instead of body mass index (BMI) because BMI was not included in the design of the Triple High Survey and because waist circumference is one of the defining criteria of metabolic syndrome, which is associated with diabetes.

## Conclusions

Increased HbA1C level was associated with increased risks of cardiovascular disease and death, the suboptimal glycemic control with HbA1c level over 7.5% (58.5 mmol/mol) was strongly associated with increased risks of stroke, coronary heart disease, and all-cause death, and the risks of ischemic stroke was increased by 1% increment of HbA1c regardless of diabetes diagnosis. We emphasize the importance of optimal glycemic control to prevent cardiovascular diseases and deaths in Taiwan.
